# Myeloid Cells in Myocardial Ischemic Injury: The Role of the Macrophage Migration Inhibitory Factor

**DOI:** 10.3390/life14080981

**Published:** 2024-08-05

**Authors:** Hao Wang, Nadiyeh Rouhi, Lily A. Slotabec, Blaise C. Seale, Changhong Wen, Fernanda Filho, Michael I. Adenawoola, Ji Li

**Affiliations:** 1 Department of Physiology and Biophysics, Mississippi Center for Heart Research, University of Mississippi Medical Center, Jackson, MS 39216, USA; hwang@umc.edu (H.W.); nrouhi@umc.edu (N.R.); lslotabec@umc.edu (L.A.S.); bseale@umc.edu (B.C.S.); cwen@umc.edu (C.W.); ffilho@umc.edu (F.F.); madenawoola@umc.edu (M.I.A.); 2 G.V. (Sonny) Montgomery VA Medical Center, Jackson, MS 39216, USA

**Keywords:** myocardial injury, macrophage migration inhibitory factor, inflammation, metabolism

## Abstract

Ischemic heart disease, manifesting as myocardial infarction (MI), remains the leading cause of death in the western world. Both ischemia and reperfusion (I/R) cause myocardial injury and result in cardiac inflammatory responses. This sterile inflammation in the myocardium consists of multiple phases, involving cell death, tissue remodeling, healing, and scar formation, modulated by various cytokines, including the macrophage migration inhibitory factor (MIF). Meanwhile, different immune cells participate in these phases, with myeloid cells acting as first responders. They migrate to the injured myocardium and regulate the initial phase of inflammation. The MIF modulates the acute inflammatory response by affecting the metabolic profile and activity of myeloid cells. This review summarizes the role of the MIF in regulating myeloid cell subsets in MI and I/R injury and discusses emerging evidence of metabolism-directed cellular inflammatory responses. Based on the multifaceted role of the MIF affecting myeloid cells in MI or I/R, the MIF can be a therapeutic target to achieve metabolic balance under pathology and alleviate inflammation in the heart.

## 1. Introduction

Myocardial injury, encompassing conditions such as myocardial infarction (MI) and I/R injury, remains a leading cause of morbidity and mortality worldwide [1]. Despite advances in medical interventions, the intricate interplay between the immune system and cardiac tissue in response to injury continues to present challenges in understanding and managing these conditions effectively. Myeloid cells, including neutrophils, monocytes, and macrophages, play pivotal roles in orchestrating the acute inflammatory response following myocardial injury, thereby influencing both the tissue damage and repair processes [2,3,4,5].

The dynamic interactions between myeloid cells and the injured myocardium are regulated by a myriad of molecular mechanisms involving various cytokines, chemokines, and signaling pathways [4,5]. Among these, the MIF emerges as a key mediator with both pro-inflammatory [6,7,8,9] and cardioprotective effects [10,11,12], depending on the context of myocardial injury. Understanding the intricate balance between the MIF and myeloid cell responses is crucial for deciphering the pathophysiology of myocardial inflammation and identifying potential therapeutic targets.

This review aims to elucidate the multifaceted roles of myeloid cells, particularly neutrophils, monocytes, and macrophages, in myocardial inflammation following injury (Table 1A). By examining the molecular mechanisms underpinning their functions and interactions, we seek to shed light on the complex immune–inflammatory pathways involved in myocardial injury and repair. Furthermore, we explore the regulatory effects of the MIF on myeloid cell responses and its implications for therapeutic interventions in cardiovascular pathologies.

Through a comprehensive analysis of the current literature and emerging research trends, this review aims to provide valuable insights into the pathogenesis of myocardial injury and potential avenues for precision interventions aimed at improving patient outcomes.

## 2. Myeloid Cell Subsets in MI or I/R

### 2.1. Cardiac Resident Myeloid Dendritic Cells (DCs)

Cardiac resident myeloid dendritic cells (DCs) bridge the innate and adaptive immune responses through antigen presentation and cytokine release [13,14]. For example, α-myosin heavy chain protein from dead cardiomyocytes can be captured by DCs and presented to T cells, initiating heart-specific immune responses that prolong inflammation and cardiac injury in the I/R model [15,16]. Similar to macrophages, DCs are heterogeneous and can be categorized based on their origins into conventional (cDC) and plasmacytoid (pDC) DCs. Both types are activated and involved in the immune response to acute myocardial infarction (MI). However, the global depletion of DCs is detrimental to the heart during MI-induced cardiac remodeling, indicating their contribution to myocardial repair [2]. Interestingly, selectively targeting one subset of DCs has shown cardioprotective effects, suggesting that some DCs might play a detrimental role in acute MI [13,17,18].

Mechanism studies on dendritic cells (DCs) highlight the activation of CD4+ T cells by exosomes derived from DCs [19] and the HMGB1/TLR4 signaling pathway [18]. Intriguingly, distinct metabolic profiles have been reported in different subsets of DCs [20], raising questions about whether metabolic alterations under pathological stress are associated with functional or phenotype changes in DCs.

To sum up, the role of dendritic cells (DCs) in myocardial inflammation induced by ischemia or reperfusion injury is quite complex. Future studies are supposed to investigate different subsets of DCs to identify their unique properties and verify their overall effects in the myocardium. Meanwhile, it will be fascinating to link the distinct metabolic profiles to any specific subsets of DCs. In this way, the association between metabolic conditions and cellular differentiation can be depicted with a strong rationale.

### 2.2. Monocytes and Macrophages

Compared to dendritic cells and neutrophils, research on mononuclear phagocytes, particularly monocytes, is more extensive, and their inflammatory response patterns to myocardial infarction (MI) and myocardial ischemia/reperfusion (I/R) are well-identified. Both monocytes and macrophages display high heterogeneity and dynamics during the acute inflammatory response to MI or I/R, participating in both injury-promoting and tissue-repairing processes. Following MI or myocardial I/R, monocytes from the peripheral blood rapidly infiltrate the ischemic region and differentiate into macrophages, supplementing or replacing cardiac resident macrophages [21]. Cardiac resident macrophages are capable of self-renewal, are relatively long-lived [22], and exhibit an M2 phenotype with anti-inflammatory properties [23]. However, only 2–5% of these resident macrophages remain in the infarct area after myocardial injury [24]. The depletion of resident macrophages results in impaired cardiac function and adverse remodeling [24].

Pro-inflammatory monocytes (Ly-6G^high^) infiltrate the injured myocardium, peaking around 3 days after MI, and have a relatively short lifespan with a pro-inflammatory secretome profile [25,26]. About 7 days after MI, anti-inflammatory and reparative M2 macrophages (Ly-6G^low^) derived from Ly-6G^high^ monocytes become the dominating subset, promoting healing and scar maturation [26,27]. A higher level of pro-inflammatory monocytes correlates with severe MI manifestations and poor cardiac function in patients [28,29]. Strategies targeting Ly-6G^high^ monocytes or M1 macrophages to alleviate the pro-inflammatory response have shown cardioprotective effects [30,31,32]. Conversely, the selective depletion of M2 macrophages increases the risk of myocardial rupture post-MI, highlighting their importance in modulating cardiac tissue healing [33]. Strategies to facilitate the polarization of M2 macrophages from Ly-6G^low^ monocytes significantly reduce the negative impact during cardiac remodeling [34,35,36]. Despite extensive research on monocyte/macrophage polarization and their roles in cardiac inflammation, the fundamental mechanisms underlying this polarization remain poorly understood.

In the past decades, our knowledge and understanding of mononuclear phagocytes have significantly expanded. Both the beneficial and detrimental functions of macrophages and monocytes in myocardial inflammation are claimed by strong evidence, but future studies should aim at suppress the detrimental effects of the mononuclear phagocytes while preserving their beneficial effects. Mechanistic studies are required to fulfill these aims and allow us to develop novel drugs that target the objects of interests.

### 2.3. Neutrophils

The primary source of neutrophils is the bone marrow. As the most abundant white blood cells, neutrophils account for 50–70% of all circulating leukocytes in humans and 10–25% in mice [37]. They act as first responders to infections and sterile inflammation. Within hours after the onset of I/R myocardial injury, a large number of neutrophils infiltrate the ischemic myocardium through the blood vessel wall from the circulating blood [4,38]. Neutrophils are attracted by damage-associated molecular pattern molecules (DAMPs) from necrotic/apoptotic cells and inflammatory mediators from adjacent cells. Recruited neutrophils help clear dead cell debris via phagocytosis [39].

Traditionally, neutrophils have been viewed as detrimental in acute MI settings. They produce reactive oxygen species (ROS), promoting a pro-inflammatory local environment that affects cardiomyocyte viability, contractility, and long-term remodeling during MI [40,41]. Neutrophil degranulation releases granular proteins such as myeloperoxidase (MPO), serine proteases, and matrix metalloproteinases (MMPs), leading to cardiac cell death and extracellular matrix (ECM) degradation. Additionally, pro-inflammatory cytokines like TNF-α, IL-1β, and chemokines (CXCL1, 2, 3 and 8) released from neutrophils can exacerbate inflammation and harm cardiomyocyte viability [42].

The formation of neutrophil extracellular traps (NETs) during NETosis contributes to microthrombosis, resulting in myocardial no-flow after I/R injury [43]. NETs also stimulate macrophage inflammasomes to release pro-inflammatory cytokines (IL-1β, IL-18) [44,45] and are linked to poorer clinical outcomes in MI patients [46,47,48,49,50]. Therapeutic strategies targeting NET formation have shown cardioprotective effects in preclinical animal models [43,49].

Conversely, recent research indicates that neutrophils also possess pro-resolving properties and can promote post-MI cardiac repair [40,51,52]. Neutrophils can acquire a pro-resolving phenotype, exhibit prolonged lifespans, and secrete pro-resolving cytokines (IL-10, TGF-β), playing a role in tissue repair [40,51,52,53]. They also release annexin A1, which promotes neutrophil apoptosis and macrophage polarization to a reparative M2 phenotype [54]. Neutrophil depletion has shown conflicting results on cardiac function in different studies, suggesting that the regulation of neutrophil responses in I/R injury remains an open question.

Meanwhile, recent evidence suggests that neutrophils play a multifaceted role in acute inflammation, participating in the resolution of inflammation, regaining tissue homeostasis, and wound healing. These beneficial effects of neutrophils have been observed in MI or myocardial I/R. Neutrophil extracellular traps (NETs), while traditionally seen as detrimental, also possess anti-inflammatory features. Neutrophil depletion in mice demonstrates the beneficial role of neutrophils in MI-induced healing [40]. Some pro-inflammatory cytokines or chemokines can be scavenged by the serine proteases inside NETs, attenuating the extent of inflammation [55,56]. NETs also promote macrophage polarization toward the reparative phenotype [57,58].

The status of neutrophils influences the content of neutrophil extracellular vehicles (EVs) and secretion, which determines their pro-inflammatory or anti-inflammatory effects [3,59]. The cell surface marker SiglecF distinguishes a novel subset of neutrophils (Ly6G^+^SiglecF^HIGH^) [60]. SiglecF^HIGH^ neutrophils appear from Day 1 after MI until Day 4 in the myocardium. These neutrophils exhibit NF-κB activation, leading to a longer lifespan within the infarct compared to the SiglecF^LOW^ subset. They also exhibit hyperactive ROS production and potentially engage in NET formation [60].

The engulfment of apoptotic neutrophils by macrophages simultaneously activates an anti-inflammatory response by inducing the production of the anti-inflammatory cytokine IL-10 and inhibiting the production of pro-inflammatory cytokines [61]. Neutrophil-secreted lipocalin mediates the expression of myeloid-epithelial-reproductive tyrosine kinase (MerTK) in macrophages, improving efferocytosis [62]. Neutrophils also stimulate the production of VEGF-A in macrophages via the receptor AnxA1 on actively infiltrating neutrophils [54]. Overall, neutrophils mediate macrophage activities and can exhibit beneficial effects in the post-infarction healing process.

Similar to the two subsets of macrophages (M1 vs. M2), neutrophils can be categorized into N1 (pro-inflammatory) and N2 (anti-inflammatory) subsets based on their distinct surface markers. In a stroke model, the number of N1 neutrophils decreased three days after cerebral infarction [63], while N2 neutrophils began to dominate as tissue repair commenced [64,65]. A pioneering study on N1/N2 neutrophils in MI reported by Ma et al. discovered that N2 neutrophils gradually accumulate in the infarcted myocardium due to the polarization of neutrophils [52]. In vitro studies revealed that DAMPs contribute to N1 neutrophil polarization while anti-inflammatory cytokines (TGFβ, IL-10, etc.) polarize neutrophils toward the N2 phenotype [52,66]. These studies underscore the potential therapeutic strategy of targeting N1/N2 neutrophil subsets in cardiac inflammation to treat MI or I/R injury.

Similar to other types of myeloid cells, neutrophils have recently been found to possess multifaceted roles in myocardial inflammation induced by ischemia and reperfusion injury. Neutrophils, as the first responders to the sterile inflammation in the myocardium, are involved in multiple processes that are associated with dead cell scavenging, local secretome formation, cell remodeling, and tissue repair. There is no doubt that neutrophils are major participants that partially determines the pattern of acute inflammatory response. With our understanding of neutrophils expanding, it will be intriguing to identify the subset of neutrophils with unique features that majorly show beneficial effects to myocardial inflammation, meanwhile suppressing the particular neutrophils that show detrimental effects.

**Table 1:** (**A**) Myeloid cell involvement in myocardial infarction (MI) and ischemia–reperfusion injury(I/R). (**B**) MIF involvement and the role of metabolic and regulatory processes in myocardial infarction (MI) and ischemia–reperfusion injury (I/R).

(A)				
Myeloid Cells	Key Processes	Mechanisms/Key Molecules	Functional Outcome	References
**Macrophages**	Inflammation, Polarization, Phagocytosis	PDK1,4, Hexokinase, 6-PFK, Succinate, OXPHOS, NAD+, TNF-α, IL-1β, IL-6	Healing, Regulation of Inflammation	[42,52,66]
**Neutrophils**	Oxidative burst, Phagocytosis, NET Formation	Ly6G+SiglecF^HIGH^, MPO, ROS, HOCl, superoxide, CXCR2, CXCR4, CXCR7, SiglecF, AnxA1, MerTK	Regulation of Inflammation	[40,42,50,62,64,67,68]
	Modulation of Macrophage Activity	Serine Proteases, MMPs, Lipocalin	Tissue Repair	
**Monocytes**	Chemotaxis, Infiltration, Differentiation	MCP-1, CCR2, CCR2, CXCR2, JNK Pathway, HIF-1α, PGC-1β, GLUT1, Ly-6G^high^	Modulation of Inflammation, Tissue Remodeling	[25,26,68,69,70]
**Dendritic Cells**	Antigen Presentation, T Cell Activation	Exosomes, HMGB1/TLR4 Signaling Pathway	Activation of CD4+ T Cells, Induction of Inflammation	[18]
**(B)**				
**Processes and Factors**	**Key Processes**	**Mechanisms/Key Molecules**	**Functional Outcome**	**References**
**MIF Involvement**	Inflammation, Cell Survival	MIF-CD74 Signaling Axis Pathway, CD74, Src Kinase, PI3K, AKT, ERK, p38 MAPK	Modulation of Inflammatory Cytokine Production (TNF-α, IL-1β) Inhibition of Apoptosis through Interaction with the CD74 Receptor Enhancement of Cellular Responses to Hypoxia and Oxidative Stress	[2,68,71,72,73,74]
**Metabolic Processes**	Energy Production, Cellular Energetics	Glycolytic Enzymes AMPK, PGC-1α, Succinate, OXPHOS	Modulation of ATP Generation via Multiple Metabolic Pathways, Regain Metabolic Homeostasis	[11,22,23,63,69,70,75,76,77,78,79]
**Regulatory Factors**	Gene Expression, Immunometabolism, Cell Signaling	NF-κB, STAT3	Cellular Adaptation, Tissue Homeostasis	[18,50,54,62,80,81]

## 3. Metabolic Regulation of Myeloid Cells in MI or I/R

Recent studies on macrophage and monocyte metabolism revealed intriguing correlations between metabolic profiles and cellular inflammatory activities. In patients with myocardial infarction (MI), pro-inflammatory monocytes exhibit increased glycolysis, which is associated with the upregulated production of pro-inflammatory cytokines, greater infarct size, and poorer cardiac systolic function [75,79]. Under ischemic conditions, the activation of macrophage HIF-1α leads to the upregulation of glucose transporters (GLUT1) and glycolytic enzymes (PDK1,4, hexokinase, 6-PFK), favoring glycolysis [11,22,23,75,76,77,78,79].

The accumulation of metabolites from the tricarboxylic acid (TCA) cycle and oxidative phosphorylation (OXPHOS) in CCR2+ macrophages is associated with changes in cellular activity. During reperfusion, succinate oxidation increases reactive oxygen species (ROS) generation, exacerbating local inflammation [69,70]. A current hypothesis suggests that pro-inflammatory cardiac macrophages predominantly metabolize glucose via glycolysis while reparative macrophages favor mitochondrial OXPHOS as their energy source [82].

Fatty acids, as a major energy substrate predominantly utilized in the heart, are also associated with macrophage phenotype changes. Reparative macrophages show increased fatty acid oxidation in the mitochondria and elevated NAD+ levels with activated PGC-1β [83]. Conversely, disruption of OXPHOS complex I is linked to increased inflammatory responses and aggravated cardiac injury after MI [84].

Given this information, it is reasonable to assume that the selective activation of a metabolic pathway or the accumulation of specific metabolites affects cellular activities and polarization under pathological stress. This metabolic regulation could be a promising therapeutic target for modulating inflammatory responses and promoting tissue repair in myocardial injury. In summary, the metabolic state of myeloid cells significantly influences their function and role in myocardial inflammation and repair. Pro-inflammatory macrophages are characterized by increased glycolysis, while reparative macrophages rely more on OXPHOS and fatty acid oxidation. Understanding and manipulating these metabolic pathways could provide new strategies to enhance cardiac repair and improve outcomes after myocardial injury.

## 4. Role of the Macrophage Migration Inhibitory Factor (MIF) in MI or I/R

The MIF is constitutively expressed in a variety of myeloid cells, including monocytes, neutrophils, macrophages, and dendritic cells. Non-immune cells such as epithelial cells, endothelial cells, stromal cells, cardiomyocytes, and fibroblast cells also express the MIF [7,85]. The MIF acts as a major regulator in myocardial injury (Table 1B), exhibiting both pro-inflammatory [7,8] and cardioprotective effects [10,11,12,86,87] depending on the duration of ischemia and the extent of myocardial injury.

### 4.1. Cardioprotective Effects of the MIF

The cardioprotective effects of the MIF are primarily attributed to its role in regulating cardiac metabolism under pathological stress. One of the key mechanisms involves the MIF binding to the CD74 receptor, which triggers downstream events, including AMPK activation [10,87]. This activation increases glucose uptake by relocating the GLUT4 transporter to the cytoplasm membrane, thus upregulating the glucose metabolism [10]. Other mechanisms contributing to the MIF’s cardioprotective effects include the inhibition of JNK signaling, reduction in apoptosis [88,89], and mitigation of cellular oxidative stress [90].

### 4.2. Pro-Inflammatory Effects of the MIF

Conversely, the MIF is associated with elevated inflammatory responses in the injured myocardium. Studies have shown that MIF deficiency in mice is linked to reduced myocardial infiltration of neutrophils and macrophages [86]. Moreover, MI mice treated with anti-MIF interventions displayed decreased inflammatory cell infiltration and a lower risk of cardiac rupture [91]. These findings indicate that while the MIF has protective metabolic effects, its pro-inflammatory actions can exacerbate myocardial damage in certain contexts.

The dual role of the MIF in myocardial injury underscores its complex function in cardiac pathology. The balance between its cardioprotective and pro-inflammatory effects is influenced by the extent and duration of ischemic injury. Understanding this balance is critical for developing targeted therapeutic strategies that leverage the MIF’s beneficial effects while mitigating its detrimental effects.

### 4.3. Future Directions of MIF Study and the Potential Therapeutic Implications

Based on up-to-date information, there are no current clinical research programs aimed at identifying the MIF or testing MIF-associated agonism/antagonism in the context of cardiovascular disease. But recently, laboratory studies revealed that the regional application of the MIF could be beneficial in the short term in the scenario of myocardial ischemia via binding to the CD74 signaling cascade. The administration of the MIF agonist or the MIF will act therapeutically to upregulate the cardioprotective metabolic adaption under ischemia or reperfusion stress and rescue the ischemic heart tissue from necrosis. Such proof-of-concept tests have been performed on the murine cardiac ischemia model, showing some cardioprotective effects. More importantly, small-molecule MIF agonists, such as MIF20, also have introduced a novel approach for enhancing MIF signaling transduction through CD74. On the other hand, compared to the recombinant proteins, small molecules possess greater target tissue infiltration and a possibility of oral administration.

## 5. Modulation of Myeloid Cell Responses by the MIF

During the early phase of the cardiac inflammatory response to MI or I/R injury, leukocytes, especially myeloid cells including granulocytes and monocytes, infiltrate the injured myocardium, playing critical roles in tissue remodeling and the healing process. The infiltration, activation, and polarization of myeloid cells are crucial for the removal of dead cells and scar tissue formation [5,92]. The activities of each type of myeloid cell follow specific temporal and spatial patterns, orchestrated by various cytokines, chemokines, toll-like receptors, and oxidative stress; and are influenced by the local microenvironment corresponding to the signaling mechanisms of myocardial injury [93].

### 5.1. The MIF and Dendritic Cells

The MIF drives the mobility of dendritic cells (DCs) from the bone marrow in a CD74-dependent manner via the activation of the Src and PI3K kinases [94]. The MIF-CD74 signaling axis mediates hepatic DC apoptosis [74]. In cancer studies, the MIF indirectly hindered the anti-tumor activity of DCs [72]. The immunosuppressive effect of the MIF-CD74 signaling axis in DCs, particularly in metastatic melanoma, has been reported in terms of the activation markers on the cell surface. The MIF also induces the phosphorylation of AKT(S473) and ERK(Thr202/Tyr204) in DCs [71]. Additionally, the MIF induces DC maturation in the context of intraperitoneal infection by activating the p38 MAPK pathway [68]. However, there are few studies on the effect of the MIF on DCs in the context of MI or myocardial I/R, highlighting a gap in understanding MIF-modulated immune responses in these conditions.

### 5.2. The MIF and Macrophage Polarization

The MIF was first discovered as a T cell-derived cytokine that inhibits macrophage migration [95,96]. It regulates M1 macrophage polarization via the CD74-CXCR2-JNK pathway in mice [97]. An MIF antagonist attenuates tumor-associated macrophage polarization toward the M2 phenotype [98]. The MIF also facilitates the conversion of macrophages into foam cells in the blood vessel wall, contributing to plaque destabilization [99,100]. However, few studies have comprehensively explored the role of the MIF in modulating macrophage activities in MI or myocardial I/R.

### 5.3. The MIF and Neutrophils

Studies on the effect of the MIF on neutrophils, particularly in MI or myocardial I/R injury, are relatively limited. The MIF increases the production of hypochlorous acid (HOCl) and superoxide in phagocytic neutrophils and induces NET formation via PMA [101]. Recent research indicated that the MIF affects NET formation via an oxidant-independent mechanism [73]. These findings reveal that the MIF has a direct effect on neutrophils through the C-X-C chemokine receptors (CXCR2, CXCR4, CXCR7) on the cell membrane, rather than CD74, which is not expressed on neutrophils [93,102,103]. The increased oxidative content production in neutrophils activated by the MIF could be associated with enhanced phagocytosis, improving cell debris scavenging in the damaged myocardium.

Moreover, the MIF inhibits neutrophil apoptosis by inducing the release of the pro-survival mediator CXCL8 from mononuclear cells via the CXCR2 receptor [104]. The MIF acts on neutrophils and mononuclear cells (monocytes or macrophages), potentially leading to indirect synergistic effects due to the co-localization of different cell types at the inflammatory site. However, the direct effects of the MIF on neutrophils remain less studied. Given that neutrophil infiltration peaks one day after myocardial injury and subsides after about three days [92], it is reasonable to conclude that studying the direct effects of the MIF on neutrophils in the context of MI or myocardial I/R is crucial for understanding the acute inflammatory response (24 h to 3 days).

## 6. Conclusions

This comprehensive review explores the intricate interplay between myeloid cells, particularly neutrophils, monocytes, and macrophages, and their critical roles in the acute inflammatory response following myocardial injury. By clarifying the multifaceted functions of these immune cells in MI and I/R injury, we have underscored their contributions to both the tissue damage and repair processes. Notably, the rapid recruitment and activation of neutrophils, coupled with the phenotypic diversity of monocytes and macrophages, orchestrates a complex immune response critical for myocardial healing.

**Figure 1:**
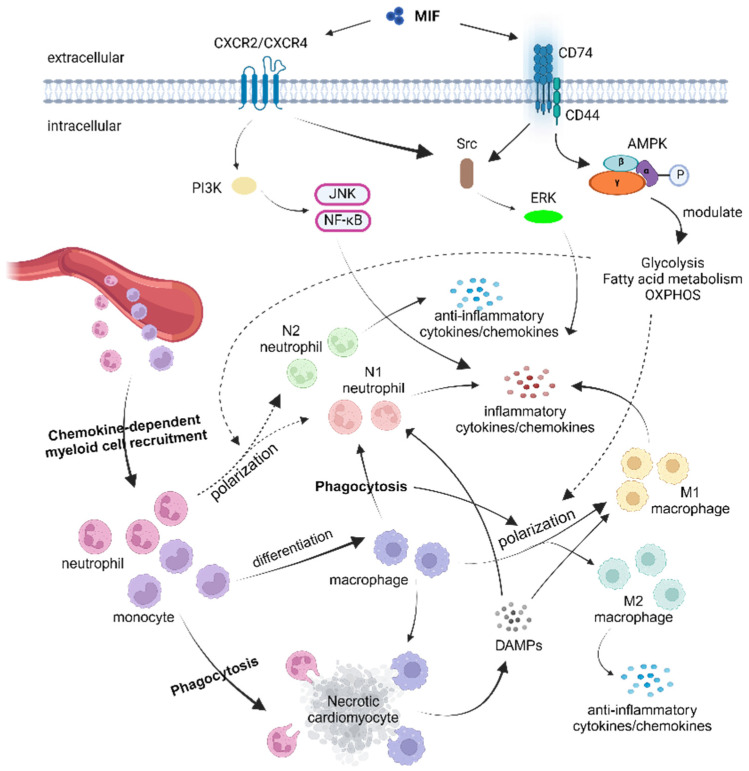
Cellular response of neutrophils, monocytes, and macrophages to ischemic myocardial injury, with emphasis on MIF-mediated regulation of metabolism and inflammatory signaling. Ischemia and reperfusion stress induce cardiac cell death. The necrotic cardiac cells release content that causes damage-associated molecule patterns locally, which attracts myeloid cell recruitment in a chemokine-dependent pattern [61]. Neutrophils, as the first responders, along with monocytes, infiltrate the injured myocardium and help to remove the necrotic cells by phagocytosis [40,41]. Meanwhile, the DAMPs and MIF-CXCR2/CXCR4 signaling trigger the production of inflammatory cytokines via the NF-kB or JNK pathway [97,104]. The inflammatory cytokines contribute to the polarization of M1 macrophages and N1 neutrophils in situ. However, MIF-CD74/CD44 signaling activates AMPK and other downstream factors [10,11,12], which might alter the cellular metabolism of myeloid cells. Meanwhile, MIF-CD74/CD44 signaling could also be associated with the production of anti-inflammatory cytokines, helping the polarization of M2 macrophage and N2 neutrophils. M2 and N2 cells are considered to have anti-inflammatory properties. Consequently, neutrophil and macrophage polarization toward the anti-inflammatory phenotype (N2 or M2) [40,52,78] will eventually benefit the long-term reparative process.

Furthermore, our exploration of the MIF sheds light on its regulatory effects on myeloid cells, highlighting its potential as a key modulator of inflammation and tissue repair in the context of myocardial injury. Despite the existing body of the literature on the MIF’s involvement in immune responses, particularly in phagocytic neutrophils and mononuclear cells, further investigations are warranted to understand the precise mechanisms by which the MIF influences myeloid cell functions during the acute phase of inflammation in MI and myocardial I/R (Figure 1).

Moving forward, future research efforts should aim to clarify the intricate crosstalk between the MIF, myeloid cells, and cardiac metabolism under pathological stress conditions. Utilizing advanced research tools, such as single-cell multi-omics technologies, holds promise for understanding the metabolic–inflammatory axis at the cellular level and identifying novel therapeutic strategies to reduce myocardial injury and promote optimal recovery. By bridging the gap between immune regulation, metabolic pathways, and inflammatory responses, we can prepare the way for precision interventions that target specific cellular interactions and pathways to enhance myocardial healing and functional outcomes in cardiovascular diseases.

This review underscores the critical importance of understanding the dynamic interplay between myeloid cells and the MIF in myocardial injury, offering a foundation for future studies aimed at understanding the complexities of immune regulation and tissue repair in cardiovascular pathologies.

## Data Availability

The original contributions presented in the current study are included in the article. Further inquiries can be directed to the corresponding author.

## Abbreviations

AMPK	AMP-activated protein kinase
DAMPs	Damage-associated molecular pattern molecules
DCs	Dendritic cells
HOCl	Hypochlorous acid
I/R	Ischemia and reperfusion
MI	Myocardial infarction
MIF	Macrophage migration inhibitory factor
NETs	Neutrophil extracellular traps
OXPHOS	Oxidative phosphorylation
